# Retentive Value and Cyclic Fatigue Resistance of Polyetheretherketone Clasps as an Alternative to Cobalt-Chrome Clasps for Removable Partial Dentures: A Systematic Review

**DOI:** 10.7759/cureus.98435

**Published:** 2025-12-04

**Authors:** Imad Al-Banyahyati, Amal Sefrioui, Salwa Berrada, Fatima Zaoui, Hassnae Benyahia

**Affiliations:** 1 Prosthodontics, Abulcasis International University of Health Sciences, Rabat, MAR; 2 Prosthodontics, Faculty of Dental Medicine, Mohammed V University in Rabat, Rabat, MAR; 3 Orthodontics and Dentofacial Orthopedics, Faculty of Dental Medicine, Mohammed V University in Rabat, Rabat, MAR

**Keywords:** clasps, co-cr, cyclic fatigue, peek material, retention

## Abstract

The aim of the review was to evaluate polyetheretherketone (PEEK) clasps used for removable partial dentures (RPDs) as alternatives to conventional cobalt-chrome (CoCr) clasps and assess their retentive potential and behavior in terms of resistance to cyclic fatigue for long-term use. This review was performed according to the Preferred Reporting Items for Systematic and Meta-Analysis (PRISMA) criteria and was registered in the International Prospective Register of Systematic Reviews (PROSPERO) (CRD42023459699). An electronic search was performed independently by two examiners in PubMed/MEDLINE and ScienceDirect databases for articles published in English between 2017 and 2023. Risk of bias was assessed using the Joanna Briggs Institute (JBI) critical appraisal tool. Initially, 92 articles were identified and subjected to screening. Among these, six articles met the eligibility criteria for further analysis. The inter-rater agreement for article selection was high (91.67% agreement, Cohen’s kappa coefficient = 0.83), with no exclusions based on risk of bias assessment. PEEK clasps, though generally exhibiting lower retention compared to CoCr, still met clinical standards. Factors affecting their performance included manufacturing, dimensions, and aging. While PEEK offers advantages in stress distribution and enamel wear reduction, concerns persist about its mechanical properties compared to CoCr, necessitating careful design considerations. Within the limitations of our systematic review, PEEK clasps may be a promising alternative to CoCr clasps in RPDs, potentially offering aesthetic benefits and reduced stress on abutment teeth. A global approach that analyzes both in vitro and in vivo studies is recommended to fully assess their performance.

## Introduction and background

Removable partial dentures (RPDs) remain a valid treatment option for managing partial edentulism despite the advent of implant-based solutions. RPDs offer minimally invasive procedures to restore aesthetics and function, with their retentive potential primarily governed by clasps [1]. Cobalt-chrome (CoCr) alloys are widely used for RPD frameworks due to their excellent mechanical properties, high retentive potential, and well-documented reproducible results [1-3]. However, they have drawbacks, such as the deterioration and fracture of retentive arms under cyclic fatigue [1-3]. A retentive clasp is subjected to repeated stresses resulting from dynamic movements in the mouth, including insertion and removal of the RPD. This aspect is crucial when assessing whether a material is suitable for making framework clasps for clinical use [1-3]. Other noted disadvantages include the exposure of clasps when the patient smiles, giving an unpleasant appearance [1-3]. Various studies have indicated that CoCr alloys can, albeit rarely, lead to sensitization, irritation, and allergic reactions despite having relatively low toxicity compared to nickel [4]. Furthermore, one should consider that the metallic nature and significant weight of these alloys might result in undesirable galvanic reactions when they come in contact with saliva, creating psychological discomfort for patients who prefer not to have metals in their mouths [5,6].

Many patients opt for metal-free restorations that do not leave any residual chemicals behind, even without specific contraindications, as they seek swift results [7]. The use of CoCr frameworks may be replaced by polyetheretherketone (PEEK), a biocompatible material with a fully digital workflow, from taking impressions to the actual fabrication using computer-aided design and computer-aided manufacturing (CAD/CAM) technology. PEEK shows promise as a material for RPDs because of its excellent mechanical properties [8]. Moreover, because of its low reactivity and inert behavior in the mouth, PEEK may offer a favorable alternative to CoCr [9,10]. This review aimed to demonstrate how PEEK clasps compare to CoCr clasps in terms of retention and resistance to cyclic fatigue in RPD rehabilitation.

## Review

Materials and methods

The study protocol was registered online in the International Prospective Register of Systematic Reviews (PROSPERO) database (CRD42023459699). To identify relevant articles addressing the research question, "What is the comparative effectiveness of PEEK clasps as an alternative to CoCr clasps in terms of retentive value and resistance to cyclic fatigue for removable partial dentures?," the population, intervention, comparison, and outcome (PICO) framework - following the Preferred Reporting Items for Systematic Reviews and Meta-Analyses (PRISMA) guidelines [11] - was defined as follows: Population (P): clasps used in RPDs; Intervention (I): use of PEEK clasps in RPDs; Comparison (C): conventional CoCr clasps for RPDs; Outcome (O): comparison of retentive value and resistance to cyclic fatigue between PEEK clasps and CoCr clasps.

The following Boolean search equation was established: "(PEEK) AND (Removable Partial Denture)", and was employed in both PubMed and ScienceDirect to identify relevant articles to the research question. PubMed utilized a more refined search string incorporating Medical Subject Headings (MeSH) terms and various field tags for "PEEK" to capture a comprehensive range of relevant terminology: ("polyetheretherketone"[Supplementary Concept] OR "polyetheretherketone"[All Fields] OR "peek"[All Fields]) AND ("denture, partial, removable"[MeSH Terms] OR ("denture"[All Fields] AND "partial"[All Fields] AND "removable"[All Fields]) OR "removable partial denture"[All Fields] OR ("removable"[All Fields] AND "partial"[All Fields] AND "denture"[All Fields])).

To minimize the risk of reviewer bias, two reviewers (HB and IA) independently conducted electronic literature searches and assessed the eligibility of studies based on predefined inclusion and exclusion criteria. Articles published between 2017 and 2023 that addressed the retention or cyclic fatigue resistance of PEEK and CoCr RPD clasps in English were included in this systematic review. Studies not addressing retention or cyclic fatigue resistance of PEEK and CoCr RPD clasps, along with those focusing on interventions beyond the scope of this review, such as fixed prostheses, implant-supported restorations, space maintainers, speech bulb prostheses, complete dentures, reduction guides, and obturators, were excluded. Search results were imported into an Excel spreadsheet (Microsoft Corp., Redmond, USA) for an initial elimination based on title and abstract. For the full-text screening, inter-rater reliability was assessed using Cohen’s kappa coefficient. Reviewers were provided with documents containing the list of articles and evaluated them independently using pen and paper. Any disagreements between the reviewers were resolved through discussion with a third reviewer until a consensus was reached. This method ensured a thorough and impartial evaluation of the included studies, enhancing the reliability and validity of the systematic review findings. Table 1 lists the filters used during the electronic database search. Data were extracted by one reviewer (IA) and examined by the other reviewer (HB). The following data were extracted from the selected articles: author, year, specimen characteristics, testing methodology, and main findings. The collected data were gathered into a structured table.

**Table 1:** Search filters used for the systematic review

PubMed	ScienceDirect
From the year 2017 to 2023	Years: 2017, 2018, 2019, 2020, 2021, 2022, 2023
	Subject area: Medicine and dentistry
	Article type: Research articles, short communications

The risk of bias in the included studies was evaluated based on the study design following the Joanna Briggs Institute (JBI) critical appraisal checklist specific to quasi-experimental studies [12]. All articles selected for inclusion in the systematic review that met the inclusion criteria described in the protocol underwent rigorous assessment by the two reviewers (IA and HB).

Results

**Figure 1:**
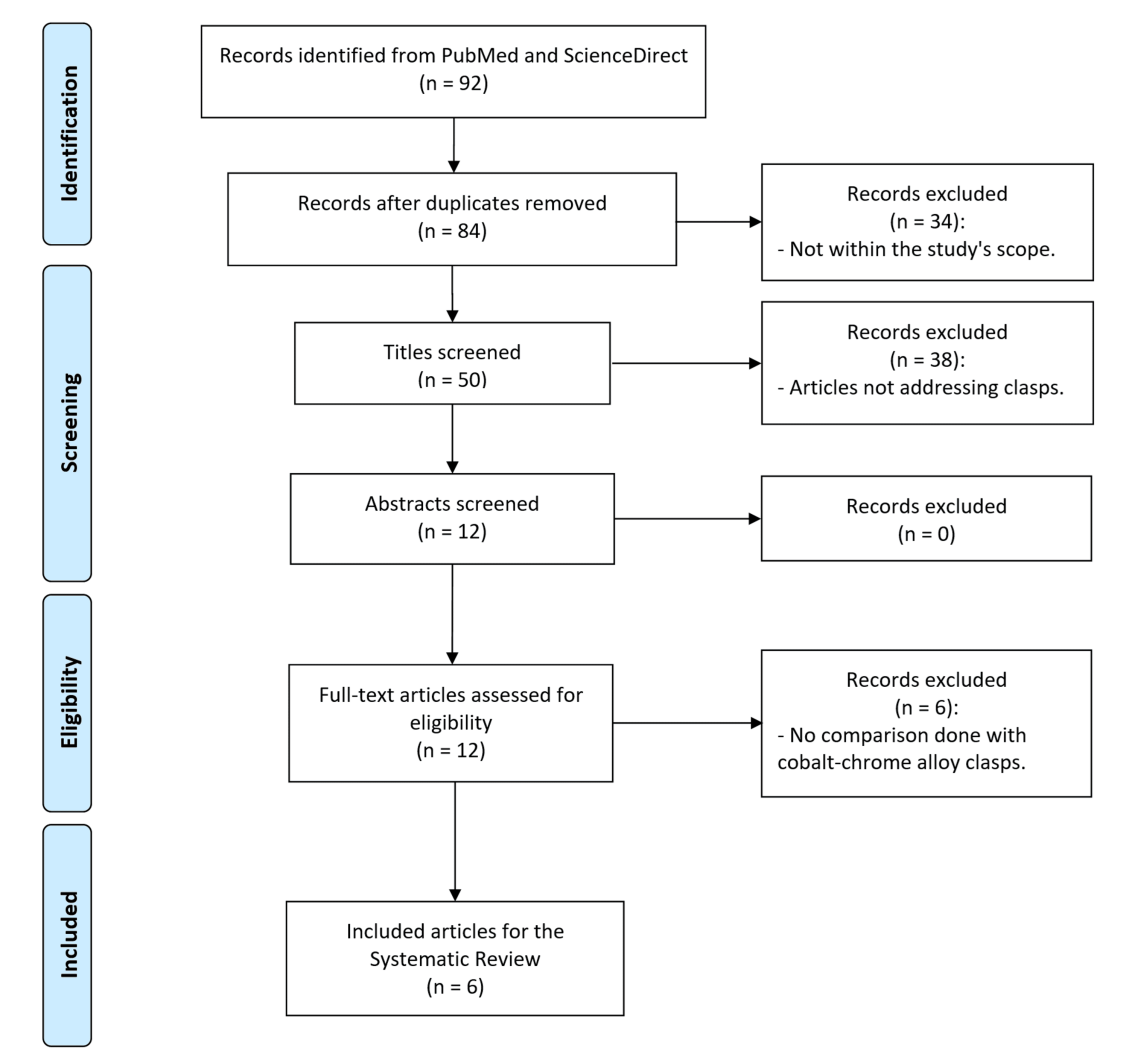
PRISMA flow diagram for the systematic review PRISMA = Preferred Reporting Items for Systematic Reviews and Meta-Analyses

Following an advanced search in the selected databases, bibliographic references were gathered, with 44 originating from PubMed and 48 from ScienceDirect. The combined raw database initially contained 92 published articles, ultimately resulting in the selection of six articles relevant to the research question (Figure 1).

During the final article selection stage, the inter-rater agreement for the assessed criteria was observed to be high, with a percentage agreement of 91.67%. Cohen’s kappa coefficient calculated for this agreement was 0.83, indicating almost perfect agreement between the raters (p < 0.001).

Upon assessing the scientific quality of the included articles using the JBI checklist, no articles were excluded, as summarized in Table 2. Questions 2, 3, and 6 of the JBI checklist did not apply to this systematic review due to the in vitro nature of the included studies. Two out of the six included articles did not meet all the checklist criteria, as both Zheng et al. and Tribst et al. studies had a low risk of bias score calculated at 83.33% while still being eligible for inclusion [1,3].

**Table 2:** Risk of bias assessment using the JBI critical appraisal checklist for quasi-experimental studies (non-randomized experimental studies) Q1-Q9 correspond to the nine JBI appraisal items [12]. Y = Yes; N = No; U = Unclear; N/A = Not Applicable; JBI = Joanna Briggs Institute

Author and year	Q1	Q2	Q3	Q4	Q5	Q6	Q7	Q8	Q9	Eligibility
Tribst et al., 2020 [1]	Y	N/A	N/A	Y	N	N/A	Y	Y	Y	5/6 (83.33%) - Included
Zheng et al., 2022 [3]	Y	N/A	N/A	Y	N	N/A	Y	Y	Y	5/6 (83.33%) - Included
Micovic et al., 2021 [13]	Y	N/A	N/A	Y	Y	N/A	Y	Y	Y	6/6 (100%) - Included
Mayinger et al., 2021 [14]	Y	N/A	N/A	Y	Y	N/A	Y	Y	Y	6/6 (100%) - Included
Gentz et al., 2022 [15]	Y	N/A	N/A	Y	Y	N/A	Y	Y	Y	6/6 (100%) - Included
Peng et al., 2020 [16]	Y	N/A	N/A	Y	Y	N/A	Y	Y	Y	6/6 (100%) - Included

The characteristics and extracted data from the six included articles are summarized in Table 3. Numerous testing methodologies have been employed by various scientific articles with the objective of studying the behavior of clasps in the context of in vitro studies to assess the suitability of PEEK clasps as replacements for CoCr clasps. To date, there is no standardized method for measuring their retention. Three out of six included articles employed insertion and removal testing methodologies [13-15]. Two others utilized finite element analysis: one conducted a constant deflection test [16], while another performed an in silico analysis [1]. The last selected article relied on fatigue testing until specimen failure [3] (Table 3).

**Table 3:** Characterstics of included studies Manufacturer details: BioHPP® (bredent GmbH & Co. KG, Senden, Germany); Dentokeep (NT-Trading GmbH & Co. KG, Karlsruhe, Germany); JUVORA (Juvora Ltd., UK; part of Victrex plc/Invibio); Ultaire® AKP (Solvay Dental 360, Alpharetta, USA); VESTAKEEP® (Evonik Industries AG, Essen, Germany). PEEK = Polyetheretherketone; CoCr = Cobalt-chromium; CoCrMo = Cobalt-chromium-molybdenum; CAD/CAM = Computer-aided design and computer-aided manufacturing; RPD = Removable partial denture; SEM = Scanning electron microscope

Author and year	Specimen characteristics	Testing methodology	Main findings
Tribst et al., 2020 [1]	Akers' clasps in PEEK, polyamide, polyoxymethylene, gold, titanium, and CoCr were modeled on virtual molars with three different undercut depths (0.25, 0.5, and 0.75), resulting in a total of 18 groups.	In silico analysis was conducted using finite element analysis on virtual 3D models created from a scanned molar. These models were developed using dedicated software to differentiate between the enamel layer, dentin thickness, and the root and periodontal ligament. The simulation was made possible by introducing into the software the specific properties of each material and dental element included in this study.	The use of PEEK is not suitable for fabricating clasps for RPDs due to the maximum stress occurring during dislodgement from significant undercuts exceeding the material’s strength. The retentive value of PEEK clasps was markedly lower than that of CoCr clasps (6.45 to 18.36 N compared to 21.78 to 65.37 N, depending on various undercuts), but CoCr clasps exerted more stress on the tooth than PEEK clasps.
Zheng et al., 2022 [3]	30 dumbbell-shaped specimens were designed using CAD/CAM to simulate the dimensions of a clasp for RPDs with different materials: 10 dumbbells in milled PEEK (JUVORA), 10 in cast CoCr, and 10 in laser-sintered CoCr.	A cantilever jig, to which the specimens were fixed with the opposite end subjected to compressive loading, facilitated simulation at different undercut depths (0.25, 0.5, and 0.75 mm) by deflection. Before measuring fatigue resistance, the mean stress applied to the specimens was initially calculated based on the degree of deflection by subjecting the specimens to fatigue cycles of 30,000 cycles or until failure. Finally, a freely rotating machine at 540 rpm, with deflection degrees simulating the predetermined undercuts, allowed the calculation of cyclic fatigue for 30,000 cycles and, if applicable, recorded the number of cycles until failure. Fractured surfaces were analyzed using an SEM.	PEEK exhibited significantly higher fatigue resistance than both cast and laser-sintered CoCr (p < 0.01), despite having significantly lower deflection resistance (p < 0.001). Simulated clasp specimens with an undercut depth of 0.25 mm demonstrated significantly higher fatigue resistance than the other groups (p < 0.001).
Micovic et al., 2021 [13]	60 Bonwill clasps with a molar undercut of 1 mm and premolar undercut of 0.75 mm, divided into four groups: 15 in pressed BioHPP® PEEK (PEEKpressed), 15 in milled Dentokeep PEEK (PEEKmilled1), 15 in milled BioHPP® PEEK (PEEKmilled2), 15 in cast CoCr (CoCrMo).	A machine performed 50 insertion and removal cycles measured on a molar and premolar model in abrasion-resistant CoCr at a rate of 5 mm/min until the maximum force required for 10% displacement was reached. This test was conducted on the clasps at different measurement intervals: at the start, after 90 days of storage in artificial saliva at 37°C, and after 180 days of storage in artificial saliva at 37°C.	The mean retention values in Newtons (95% CI) for each group: PEEKmilled1 (Dentokeep): Start: -1.7 (-3.4, -0.02); 90 days: -9.1 (-9.9, -8.2); 180 days: -0.7 (-1.2, -0.2). PEEKmilled2 (BioHPP®): Start: 7.5 (6.4, 8.6) (increasing); 90 days: -0.5 (-1.2, -0.2); 180 days: -6.4 (-7.3, -5.5). PEEKpressed: Start: -4.8 (-6.0, -3.5); 90 days: -1.3 (-1.8, -0.7); 180 days: -4.3 (-5.0, -3.6). CoCrMo: Start: 14.8 (11.3, 18.3) (increasing); 90 days: -11.8 (-14.4, -9.1); 180 days: -6.8 (-8.7, -4.8).
Mayinger et al., 2021 [14]	60 Bonwill clasps with a molar undercut of 1 mm and premolar undercut of 0.75 mm, divided into four groups: 15 in pressed BioHPP® PEEK (PEEKpressed), 15 in milled Dentokeep PEEK (PEEKmilled1), 15 in milled BioHPP® PEEK (PEEKmilled2), 15 in cast CoCr (CoCrMo).	A machine performed 50 insertion and removal cycles measured on a molar and premolar model in abrasion-resistant CoCr at a rate of 5 mm/min until the maximum force required for 10% displacement was reached. This test was conducted on the clasps at different measurement intervals: at the start, after 30 days of storage in distilled water using an incubator at 37°C and subjection to 10,000 thermal cycles from 5°C to 55°C for 20 sec, and after 60 days of storage in distilled water using an incubator at 37°C and subjection to 20,000 thermal cycles from 5°C to 55°C for 20 sec.	The mean retention values in Newtons (95% CI) for each group: PEEKmilled1 (Dentokeep): Start: 9.5 N (0.0, 18.5), p = 0.04; 30 days: no significant difference; 60 days: 6.1 N (5.4, 6.7). PEEKmilled2 (BioHPP®): Start: -2.9 N (-4.3, -1.5), p < 0.001 (declining); 30 days: no significant difference; 60 days: 13.6 N (13.0, 14.3). PEEKpressed: Start: -3.1 N (-4.3, -2.0), p < 0.001 (declining); 30 days: 2.9 N (2.2, 3.6), p < 0.001 (the only one showing growth); 60 days: 5.0 N (4.5, 5.6). CoCrMo: Start: 11.2 N (8.9, 13.4), p < 0.001; 30 days: no significant difference; 60 days: 18.8 N (17.3, 20.4), p < 0.001.
Gentz et al., 2022 [15]	48 Akers' clasps for mandibular molars with an undercut of 0.25 mm, including: 16 Ultaire® AKP milled PEEK clasps, 16 cast CoCr clasps, and 16 milled PEEK clasps.	The clasps underwent an insertion and dislodgment simulation using a device set at a constant speed of 8 mm/sec on an artificial CoCr molar model for 15,000 cycles. The retentive value of the clasps was measured every 1,500 cycles.	Total mean retention value in Newtons: CoCr: 11.98 N; PEEK: 2.74 N.
Peng et al., 2020 [16]	3 PEEK (VESTAKEEP®) clasp arms with 0.25 and 0.5 mm undercuts, and one CoCr clasp arm with a 0.25 mm undercut.	72 rod-shaped 3D models were designed using dedicated software to simulate a clasp arm based on four width/thickness ratios, three base widths, and six different tapers. Finite element analysis enabled the selection of optimal dimensions suitable for fabricating PEEK clasps, considering average compressive load and stress distribution at 0.25 and 0.5 mm of undercuts. The three chosen models were then manufactured using PEEK (VESTAKEEP®), in addition to a CoCr rod. The four rods underwent a cyclic fatigue test on a machine for 15,000 cycles, simulating undercuts of 0.25 mm and 0.5 mm for PEEK rods and 0.25 mm for CoCr. Undercut values were maintained throughout the fatigue test by applying a sinusoidal wave frequency of 5 Hz at the rod’s loading point. The load values were recorded to analyze deformation during the test, and the specimens’ deformation in the direction of the applied force was observed using an SEM every 3000 cycles.	The models with the most optimal shape after finite element analysis are those with a taper ratio of 0.5, 0.7, and 0.9 between the base and the tip, while being the thinnest, most slender, uniformly tapered, and providing a retention value exceeding 1.6 N, which is the lowest acceptable retention force for a clinical situation according to the study. Von Mises stress analyses showed that the maximum stress concentrations were consistently located at the base of each model, with a significant difference between maximum stress values for variable transverse dimensions and taper ratios (p < 0.05), and a strong positive correlation between thickness (r > 0.8) and stress concentration. The average retention values of PEEK clasps (2.06 to 3.67 N) were lower than those of the CoCr alloy (8.26 N) for 15,000 cycles (p < 0.05). However, PEEK clasps could provide sufficient retention for clinical use. No significant difference (p = 0.11) in long-term deformation between the two materials was observed after 15,000 cycles: PEEK: mean between 0.011 and 0.017 mm; CoCr: mean of 0.017 mm.

Discussion

This review highlights that CoCr clasps exhibit higher initial retention values compared to PEEK clasps across Micovic et al., Mayinger et al., and Gentz et al. studies [13-15]. However, PEEK clasps demonstrated sufficient retention for clinical use in specific scenarios, such as posterior teeth with deeper undercuts.

To prevent the inadvertent removal of RPDs while chewing sticky foods, some studies recommend aiming for retention values ranging from 5 N to 10 N per clasp with caution [17,18]. There is no unanimous consensus on the minimum required value per clasp, as different authors propose varying values [19,20]. While determining the minimum value for clinically acceptable clasp retention is crucial, attention should also be paid to the maximum value to avoid potential adverse effects on the periodontal tissue of the tooth receiving the clasp or the risk of clasp fracture. Therefore, careful consideration during the design phase is essential [21]. To prevent trauma to the periodontal ligament, which may occur at 10 N, some authors recommend maintaining clasp retention values between 3 N and 5 N [22], while others argue that a force value of 2.3 N is sufficient for clasp retention [23]. Frank and Nicholls suggested that a retention force of 2.94 N to 7.35 N is necessary for any clasp in the case of a distal extension for a terminal edentulous area [24], preventing dislodgment during functional movements like chewing. Ahmad et al. affirmed that guide planes also provide some retentive force through friction, with an average value of 2.41 N [25], aiming to reduce the number of clasps on an RPD. The study by Peng et al., selected as part of this systematic review on optimizing the mechanical properties of PEEK clasps [16], appears to operate with a clinically required minimum retention value well below the values already described, precisely 1.60 N per clasp [19,20]. Another study by Hussein in 2022 supports this result by finding a retention value ranging between 1.68 N and 3.70 N for a PEEK clasp with a 0.75 mm undercut [21].

PEEK clasps pressed from granules are more susceptible to external influences and handling errors, as this manufacturing process involves more complex steps, including coating, heating, and cooling the mold, the actual pressure of the heated material under vacuum, and the finishing and polishing step by air abrasion [26]. Unlike BioHPP® granules (bredent GmbH & Co. KG, Senden, Germany), BioHPP® discs (bredent GmbH & Co. KG) undergo an industrial pre-pressing that could contribute to the increase in the mechanical properties of the final commercialized product [26]. On the other hand, Mayinger et al. [14] emphasize that the significant difference between the values of (PEEKmilled1) and (PEEKmilled2) could be explained by variations in the industrial production process of discs from two different brands. However, after artificial aging, the results of the two groups aligned. In a study conducted in 2021, El Mekawy and Elgamal found that PEEK clasps manufactured by the compression molding technique constituted a more effective procedure in terms of retentive power compared to the milling method with a 0.50 mm undercut and a clasp thickness of 1.50 mm [27]. It is worth noting that the two studied clasp groups were made from materials of the same brand (BioHPP®; bredent GmbH & Co. KG), but no aging protocol was conducted in this study. The differences in the chosen undercut depths across studies are important and may explain the varying results, making it challenging to reach a clear conclusion. The variability in testing conditions, such as artificial saliva versus distilled water and differences in aging protocols, likely contributed to the discrepancies in retention values observed between studies. Future research should standardize these parameters to facilitate meaningful comparisons [27].

The average tensile strength of PEEK varies from 75 MPa to 220 MPa when manufactured by injection molding, depending on its composition and mold temperature [28]. In contrast, tests show an average tensile strength of 73 MPa for PEEK manufactured using extrusion-based 3D printing. The lesser mechanical properties of PEEK parts manufactured by extrusion compared to those fabricated by injection molding may be related to poor bonding between layers, lower exuded compaction density, and the presence of voids [28].

CoCr clasps in the study by Micovic et al. experienced a considerable decrease in retention value due to artificial aging in artificial saliva. While the retention values of the three PEEK clasp groups also showed a decline, quantifying the impact of this aging process proved challenging due to the absence of significant differences among their various retention values as they remained relatively stable [13].

The average retention value of CoCr clasps remained relatively stable throughout the aging process in distilled water in Mayinger et al.’s study, unlike the results of Micovic et al.’s study, which used artificial saliva. This finding confirms the influence of artificial saliva on the CoCr group, as observed in Micovic et al.’s study [13,14]. The alloy underwent corrosion from saliva, which has been previously reported to lead to a reduction in the fatigue strength of CoCr [29]. Meanwhile, studies suggest that PEEK demonstrates superior corrosion resistance and favorable biological properties compared to other materials, making it a viable option for clinical applications. These properties include a reduced tendency for dental plaque accumulation, minimized bacterial adhesion, and a lower risk of enamel demineralization on surfaces in contact with clasps made from this material [10]. Research indicates that PEEK performs well in saliva, recommending its use in such an environment [30,31].

Prechtel et al.’s 2020 study supports the observed decrease in mean retention values of milled PEEK specimens after artificial aging, attributing it to reduced martensitic properties due to hydrothermal cycles [32]. The phenomenon of the initial abrupt increase in retention values recorded in the studies could be explained either by the abrasion of the clasps, eliminating imperfections at the beginning of insertion and removal cycles, leading to improved fit between the abutment tooth and the inner surface of the clasp, and consequently, enhanced retention, or by the creation of a rough surface due to slight abrasion of the CoCr models used in the study, resulting in friction generation [13-15,33]. A clinical study report describes the absence of wear phenomena on ceramic or enamel surfaces using PEEK clasps, commonly observed with CoCr clasps [34].

In Mayinger et al.’s study, the relatively significant decrease in retention values of PEEK clasps compared to CoCr overall could be explained by the cyclic fatigue phenomenon due to the low elastic modulus of PEEK [14]. The elastic modulus of a material should always be considered in tests involving cyclic fatigue. Materials with a high elastic modulus can regain their original shape without permanent deformation. CoCr is a rigid material with a high elastic modulus of up to 220 GPa [1]. It should, in theory, be less prone to decreases in retention caused by deformations due to repeated insertion and disinsertion compared to PEEK clasps with an elastic modulus of only about 4 GPa and a yield strength of approximately 110 MPa [1]. If a material’s yield strength is exceeded, it will enter the plastic deformation phase, decreasing retention for PEEK clasps. Therefore, the viscoelastic nature of polymers could be behind this gradual decrease in the retentive capacity of clasp arms, but their low stiffness could imply increased flexibility, allowing the exploitation of deep undercuts and leading to increased retention forces. For CoCr clasps to ensure sufficient retention, they must operate close to their yield strength. Hence, they must be designed to have a single path of insertion and disinsertion so that the clasp arms move only the "correct" depth of the undercut on the supporting tooth. Any deviation of the prosthesis along a trajectory not designed by the dentist during fabrication will result in plastic deformation of the metal, which will harden and eventually lead to the fracture of CoCr clasp arms [3,15,21,35,36].

Concerning the clinical implications of the hypothesis proposing a relationship between the elastic modulus of a clasp material and its performance in terms of retention, posterior teeth with deeper undercuts than anterior teeth could be the preferred indication for a PEEK clasp [14]. Another clinical implication of this hypothesis lies in the possibility of recommending PEEK clasps for teeth with periodontal problems and, consequently, an unfavorable prognosis, limiting the application of excessive forces that may further harm the tooth due to its limited ability to resist stresses [37].

Due to its deformable nature and elastic modulus resembling that of dentine, PEEK has the potential to offer a more even distribution of stress to tooth structures than metal alloys. This characteristic can result in a decreased transfer of stress on both the abutment teeth and the cementation interface compared to traditional materials [7]. A 2022 study by Abed and Al-Omari suggests recommending PEEK clasps with undercuts of 0.5 and 0.75 mm for teeth with malpositions, such as distoversion and mesioversion, as well as teeth with weak periodontal support that contraindicate the use of CoCr clasps [38]. Like Gentz et al., the study by Abed and Al-Omari has a limitation: it was conducted in a dry environment, without natural or artificial saliva, which could influence the retention force [15,38].

Acceptance of PEEK clasps varies across studies. While some authors like Peng et al. and Tannous et al. are optimistic about their potential, Tribst et al. find that PEEK clasps are too fragile [1,16,33]. In their study, Tribst et al. observed that a CoCr clasp with a 0.25 mm undercut has a retention force of about 21 N, whereas the same clasp in PEEK material has a significantly lower retentive value of around 6 N. If these values seem higher than those described in previous studies, it could be attributed to the in silico nature of Tribst et al.’s study. The values and figures found in this study should only be compared with each other and with other values within the same study due to the in silico method used, involving data input into dedicated digital simulation software. Therefore, it is not recommended to compare these raw values with those from other in vivo or in vitro studies [1]. As mentioned earlier, deeper undercuts could be utilized to increase the retention value, especially in the case of flexible materials like PEEK, helping to reduce the stresses applied to the tooth. A 0.75 undercut for a PEEK clasp has a retentive value of 18 N, which is 14% lower than a CoCr clasp with a 0.25 mm undercut [1]. Tribst et al. suggest that the results of their study indicate a lower risk of enamel wear and deterioration when using a PEEK clasp instead of a CoCr clasp with similar undercut depths [1].

PEEK clasps in Zheng et al.’s study exhibited lower flexural strength and retention value than the cast and laser-sintered CoCr clasps [3]. These findings align with the observations from numerous studies discussed earlier regarding the retentive value of PEEK compared to CoCr [13-15,33].​​​​​ However, El-Baz et al.’s 2020 study suggests that there might not be a considerable difference in retention between PEEK and CoCr clasps with a thickness of 1 mm at an undercut depth of 0.50 mm [39]. Nevertheless, Abd-Elrahman et al. in a 2016 study stated that "PEEK clasps should have a greater thickness than 1.0 mm (in cross-sectional diameter) when engaging a deeper undercut (0.50 mm) to gain the stiffness of a 1 mm cross-sectional diameter cast CoCr clasps, achieving clinically acceptable retention" [38-40]. To address the issue of insufficient rigidity encountered by PEEK clasps due to their low elastic modulus, which causes a decrease in retentive force as observed in the studies of Mayinger et al. and Micovic et al. [13,14], one potential solution is to manufacture these clasps with greater thickness and design them with a deeper undercut to provide adequate retention.

According to data obtained in a study conducted by Muhsin et al. in 2018 [41],​​​​​ PEEK clasps with a thickness of 1.5 mm exhibited significantly higher retention values than CoCr clasps with a thickness of 1 mm. Indeed, the highest retention was observed for PEEK clasps with a thickness of 1.5 mm and an undercut of 0.75 mm, followed by an undercut of 0.50 mm, thus confirming that PEEK clasps could achieve clinically acceptable retentive values with different dimensions compared to CoCr clasps, even after three years of simulated in vitro use [41]. Tannous et al. in a 2012 study support this outcome, albeit without evaluating an undercut of 0.75 mm. They confirm that the most promising retentive potential for PEEK clasps was in 1.5 mm thick clasps designed with a 0.50 mm undercut [33]. Gentz et al.’s study chose to manufacture clasps with an undercut of approximately 0.25 mm, a value commonly indicated for CoCr clasps. This choice resulted in lower retention values that fell below the required criteria, rendering it unsuitable for use in RPDs [15].

In Peng et al.’s study, the maximum stress concentration for all models was located at their base, a finding supported by similar studies [16]. The three groups of clasps with different shapes selected at the end of the analysis for PEEK specimen fabrication had tapers of 0.5, 0.7, and 0.9. All three selected specimens demonstrated a retention value exceeding 1.6 N, which is considered the minimum necessary value according to some studies [19,20], and they were not subject to plastic deformation, with stress values decreasing as the taper ratio decreased. Following repetitive loading, polymeric materials undergo low-cycle fatigue, leading to cyclic softening and a progressive decline in resistance to deformation, consequently leading to a gradual augmentation in deformation [42]. However, both PEEK and CoCr clasps only experienced slight deformation, and there was no significant difference between the two materials after fatigue testing. Peng et al. concluded within the scope of his study that PEEK specimens with a width of 3.00 mm, a thickness of 2.25 mm at their base, a taper ratio of 0.5, and an undercut of 0.50 mm exhibited the best mechanical properties, disregarding the typically curved and curvilinear shape of clasps and the various insertion paths required by real clinical situations that could produce greater stresses on abutment teeth, leading to permanent clasp deformation over a short period [16].

A study conducted by Güleryüz et al. in 2021 supports these findings [23]. After confirming that the measured values after aging were within clinically acceptable limits, it was found that increasing the thickness and undercut of the clasps reduced the extent of dimensional deformations of the clasps used in this study. Regarding retention force and dimensional changes, the thickness of clasps proved to be more effective than the depth of undercuts [23].

Zheng et al. also confirm the interesting fatigue resistance reported for PEEK compared to cast and laser-sintered CoCr, with results not previously mentioned in published studies. About 94% of the PEEK specimens experienced minimal to no fractures during the simulated 30,000 cycles, leaving a negligible percentage of 6% fractures [3]. While this result may seem optimistic, it must be considered in the context of other findings highlighting the insufficient retention force of milled PEEK compared to cast and laser-sintered CoCr. This insufficient retention is a clinically relevant issue as patients may experience functional difficulties with non-retentive prostheses. The considerations mentioned above, such as dimensions and thickness, should be taken into account to ensure a clinically acceptable clasp retention value [3].

## Conclusions

Within the limitations of our systematic review, PEEK clasps may be a promising alternative to CoCr clasps in RPDs, offering aesthetic benefits and reduced stress on abutment teeth. Despite their slightly lower retentive value, this limitation can be addressed through design adjustments, while their enhanced fracture resistance remains an advantage. Their lower elastic modulus contributes to flexibility, making them suitable for cases where CoCr clasps are contraindicated, such as abutment teeth with weak periodontal support. To fully assess their performance, a global approach that includes both in vitro and in vivo studies is recommended.
